# Development and psychometric evaluation of the Decision Tool Anxiety Disorders, OCD and PTSD (DTAOP): Facilitating the early detection of patients in need of highly specialized care

**DOI:** 10.1371/journal.pone.0256384

**Published:** 2021-08-19

**Authors:** Frédérique C. W. van Krugten, Meriam Kaddouri, Maartje Goorden, Anton J. L. M. van Balkom, Ed W. Berretty, Daniëlle C. Cath, Gert-Jan Hendriks, Suzy J. M. A. Matthijssen, Henny A. D. Visser, Irene M. van Vliet, Werner B. F. Brouwer, Leona Hakkaart-van Roijen

**Affiliations:** 1 Erasmus School of Health Policy & Management, Erasmus University Rotterdam, Rotterdam, The Netherlands; 2 Department of Psychiatry, Amsterdam UMC, Amsterdam, The Netherlands; 3 Amsterdam Public Health Research Institute, Amsterdam, The Netherlands; 4 Academic Outpatient Clinic for Anxiety Disorders GGZ InGeest, Amsterdam, The Netherlands; 5 Outpatient Clinic for Anxiety Disorders PsyQ, The Hague, The Netherlands; 6 Department of Clinical Psychology, Utrecht University, Utrecht, The Netherlands; 7 Department of Psychiatry, University Medical Center Groningen, Groningen, The Netherlands; 8 GGZ Drenthe Mental Health Institute, Assen, The Netherlands; 9 Overwaal, Center of Expertise for Anxiety, OCD, and PTSD, Institute for Integrated Mental Health Care Pro Persona, Nijmegen, The Netherlands; 10 Behavioural Science Institute, Radboud University, Nijmegen, The Netherlands; 11 Department of Psychiatry, Radboud University Medical Center, Nijmegen, The Netherlands; 12 Altrecht Academic Anxiety Center, Altrecht GGZ, Utrecht, The Netherlands; 13 RINO Groep, Utrecht, The Netherlands; 14 Marina de Wolfcentrum, Mental Health Care Institute GGZ Centraal, Ermelo, The Netherlands; 15 Department of Psychiatry, Leiden University Medical Center, Leiden, The Netherlands; Medical University of Vienna, AUSTRIA

## Abstract

**Background:**

Early identification of patients with an anxiety disorder, obsessive-compulsive disorder (OCD), or post-traumatic stress disorder (PTSD) in need of highly specialized care could facilitate the selection of the optimal initial treatment in these patients. This paper describes the development and psychometric evaluation of the Decision Tool Anxiety Disorders, OCD and PTSD (DTAOP), which aims to aid clinicians in the early identification of patients with an anxiety disorder, OCD, or PTSD in need of highly specialized mental healthcare.

**Methods:**

A systematic literature review and a concept mapping procedure were carried out to inform the development of the DTAOP. To evaluate the psychometric properties of the DTAOP, a cross-sectional study in 454 patients with a DSM-IV-TR anxiety disorder was carried out. Feasibility was evaluated by the completion time and the content clarity of the DTAOP. Inter-rater reliability was assessed in a subsample of 87 patients. Spearman’s rank correlation coefficients between the DTAOP and EuroQol five-dimensional questionnaire (EQ-5D-5L) scores were computed to examine the convergent validity. Criterion validity was assessed against independent clinical judgments made by clinicians.

**Results:**

The average time required to complete the eight-item DTAOP was 4.6 min and the total DTAOP was evaluated as clear in the majority (93%) of the evaluations. Krippendorff’s alpha estimates ranged from 0.427 to 0.839. Based on the qualitative feedback, item wording and instructions were improved. As hypothesized, the DTAOP correlated negatively with EQ-5D-5L scores. The area under the curve was 0.826 and the cut-off score of ≥4 optimized sensitivity (70%) and specificity (71%).

**Conclusions:**

The DTAOP demonstrated excellent feasibility and good validity, but weak inter-rater reliability. Based on the qualitative feedback and reliability estimates, revisions and refinements of the wording and instructions were made, resulting in the final version of the DTAOP.

## Introduction

Although there is compelling evidence supporting the efficacy of psychological interventions in treating anxiety disorders, obsessive-compulsive disorders (OCD), and post-traumatic stress disorders (PTSD) [1–3], not all patients need and benefit from the same type and intensity of intervention [4]. In daily clinical practice, clinicians are faced with the challenge of providing the right treatment to the right patient at the right time and in the right place. The importance of this challenge is emphasised by the high demand for mental healthcare, relative to available resources, making it important to improve the cost-effectiveness of treatment decisions [5].

Although most patients with an anxiety disorder, OCD, or PTSD can and should be treated within primary care or secondary mental health services, a subset of patients requires additional expertise and support from highly specialized (i.e. tertiary) mental healthcare services [5]. Highly specialized mental health services are the services provided by highly trained mental health specialists to patients with complex mental health problems that cannot be fulfilled by primary and secondary mental healthcare services [6, 7]. Since delay in establishing the optimal treatment (intensity) has been associated with partial recovery and chronicity [8–10], early intensive treatment of patients who are predictively in need of highly specialized care is likely to reduce the treatment steps needed to achieve an adequate treatment benefit and prevent quality of life deterioration. This in turn may benefit the clinical and cost-effectiveness of treatments.

Although direct referral of patients with a severe and complex anxiety disorder, OCD, or PTSD to highly specialized mental health services may facilitate prompt, effective and efficient treatment, the effectiveness of this approach is highly dependent on the ability to identify these patients. Several measures to screen for anxiety disorders, OCD, and PTSD and assess their severity are available [11], yet psychometrically sound measures that aid the early identification of patients with a need for highly specialized care are lacking. Ideally, systematic and standardized pre-treatment assessments that not only capture the severity of the anxiety disorder, OCD, or PTSD itself, but also the complexity of the patient’s overall clinical picture should be used to aid clinicians in matching the intensity of treatment to the individual patient needs [12]. Recognizing this gap, we report on the development and psychometric evaluation of the Decision Tool Anxiety Disorders, OCD and PTSD (DTAOP). The DTAOP is an eight-item clinician-administered instrument designed to enhance the systematic and standardized early identification of patients with an anxiety disorder, OCD, or PTSD in need of highly specialized care during the diagnostic phase after referral.

## Materials and methods

### Phases of development

**Fig 1:**
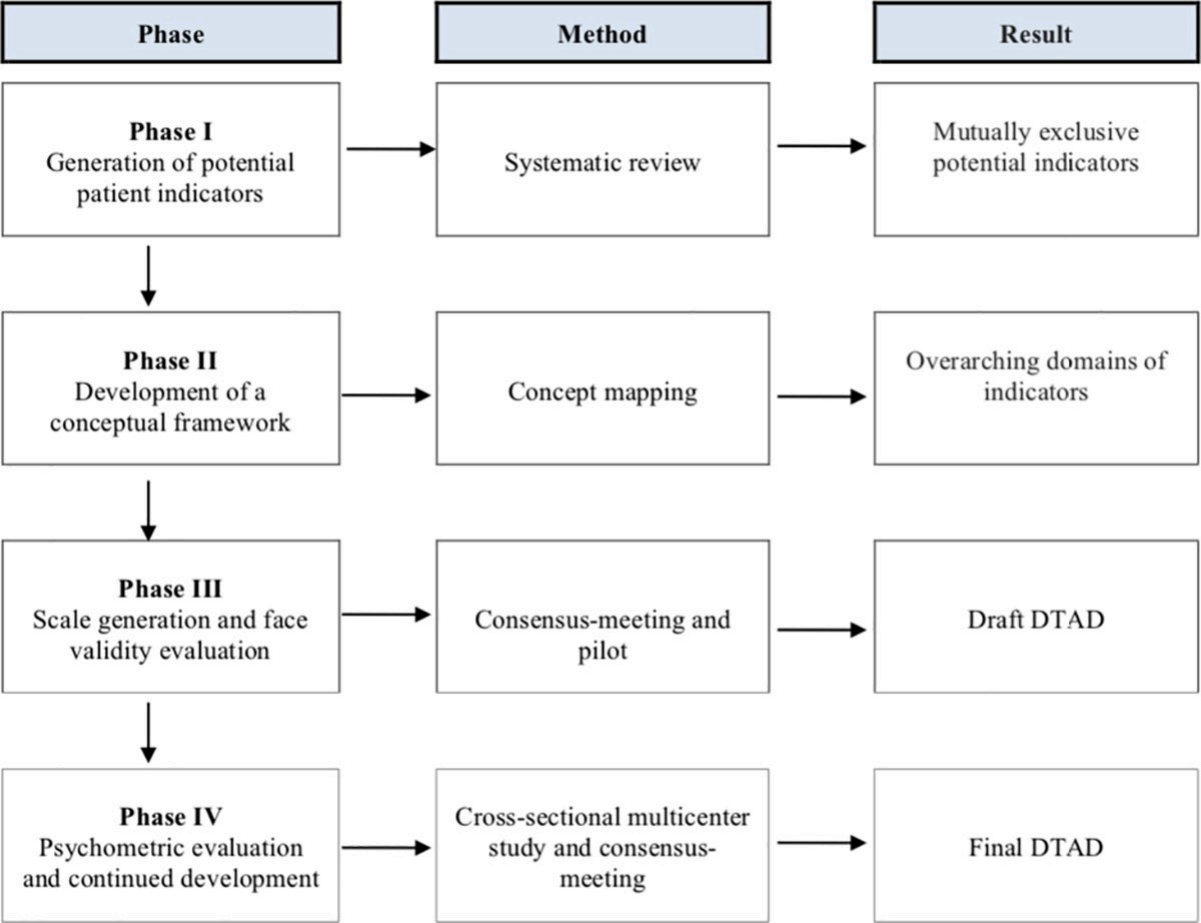
Development of the Decision Tool Anxiety Disorders, OCD and PTSD (DTAOP).

The DTAOP was initially designed for use in specialized (i.e. secondary) mental healthcare centers to inform referral decisions to highly specialized (i.e. tertiary) mental healthcare centers. The development process of the DTAOP consisted of the following four consecutive phases: (I) generation of potential indicators of patients with an anxiety disorder, OCD, or PTSD in need of highly specialized care; (II) development of a conceptual framework to guide item generation; (III) scale generation and assessment of face validity; and (IV) evaluation of psychometric properties and continued development. See Fig 1 for a visual representation of the phases. Each phase was carried out in a manner consistent with previous research on Decision Tool development (see Van Krugten et al. [13] and Van Krugten et al. [14] for more details). See below for a summary of each of the four phases. The institutional Ethical Review Committee of the Erasmus University Medical Center Rotterdam, The Netherlands reviewed the study and declared that the Medical Research Involving Human Subjects Act (WMO) did not apply to this study and that therefore an official approval by the Ethical Review Committee was not required (MEC-2016-189). Written informed consent was obtained from all participants.

Throughout each phase of the development and psychometric evaluation process, clinicians and patients were consulted. Final decisions were made by the Decision Tool Anxiety Disorders, OCD and PTSD (DTAOP) consortium, comprising 14 Dutch clinicians in the field of anxiety disorders, OCD, and/or PTSD 4 academics, and 1 patient representative.

### Generation of potential patient indicators (Phase I)

As a first step in the development process, a systematic literature review was conducted to generate a list of potential indicators for the early detection of patients with an anxiety disorder, OCD, or PTSD likely in need of highly specialized care. Following the PRISMA guidelines [15], the PubMed and PsycINFO databases were searched for primary studies published between January 2000 through April 2015 reporting potential indicators of patients with an anxiety disorder, OCD, or PTSD in need of highly specialized care. In order to identify relevant search terms, 127 clinicians and 326 patients (aged 18–75 years) were invited to participate in a web-based survey; 99 and 231 participated respectively. In the survey, participants were asked to submit search terms to identify research articles reporting indicators of need for highly specialized care. Based on the submitted terms, search strategies were composed (see S1 Appendix for the search strategies). Two reviewers independently screened the titles and abstracts of all identified references, reviewed the full texts of potentially eligible articles, and performed data abstraction using a structured, Excel-based form [16]. Based on the abstracted data, a list of potential indicators of patients with an anxiety disorder, OCD, or PTSD in need of highly specialized care was generated. No systematic review protocol was registered.

### Development of a conceptual framework (Phase II)

In phase II, a concept mapping study [17] was carried out to inform the generation of scale items. Concept mapping is a mixed-method participatory approach that integrates conventional qualitative group processes (e.g. brainstorming, pile sorting) with multivariate statistical methods of multidimensional scaling in order to depict the composite thinking of participants in a single visual framework ("the concept map"). Experts (clinicians and researchers) in the field of anxiety disorders, OCD, and/or PTSD were invited to participate in the concept mapping process. In total, 147 experts were approached, 34 of which participated in the subsequent stages of the concept mapping procedure (i.e. brainstorming and sorting). The concept mapping process and the subsequent data analyses were carried out using Concept Systems software (Concept Systems Incorporated, Ithaca, New York). During the brainstorming stage, participants were asked to review and, when necessary, add additional patient indicators to the list of indicators from the systematic review. Subsequently, participants were asked to sort the resulting list of indicators into conceptual categories. In order to generate preliminary cluster solutions, the sorting data were analysed using non-metric multidimensional scaling and agglomerative hierarchical cluster analyses. Following procedures recommended by Trochim [18], preliminary cluster solutions were evaluated for their within-cluster coherence of content. In a three-hour consensus meeting, members of the DTAOP consortium were asked to review cluster maps sequentially and select the optimal cluster map solution through an iterative process. The optimal concept map consisted of eight clusters, which, in their turn, consisted of individual potential indicators that jointly contribute to an overarching conceptual domain. For more details on the methods of Phase II see Van Krugten et al. [14].

### Scale generation and evaluation of face validity (Phase III)

Based on the resulting overarching domains (i.e. clusters) of indicators from the concept mapping study, consortium members constructed the draft Decision Tool in a three-hour consensus meeting. Since the aim was to develop an easily administrable measure, each of the overarching domains was operationalized into a dichotomous (absent/present) item, resulting in an eight-item draft version of the DTAOP. Response options of the draft DTAOP are "Yes" and "No", scored as 1 and 0, respectively. To ensure face validity, the draft DTAOP was pilot-tested in a sample of 25 outpatients aged 18 years and over with a primary anxiety disorder according to Diagnostic and Statistical Manual of Mental Disorders (DSM)-IV-TR criteria [19]. Clinicians were asked to administer the DTAOP, indicate whether the DTAOP was complete and useful and provide qualitative comments on item clarity. Based on the pilot data, minor changes were made in the wording of some of the items to ensure item clarity.

Note that at the time of data collection, the DSM-IV-TR [19] was in use in The Netherlands, in which obsessive-compulsive disorder (OCD) and post-traumatic stress disorder (PTSD) are classified as anxiety disorders. The DSM-5 [20] chapter on anxiety disorders, however, no longer includes OCD and PTSD. To accommodate future use of the DTAOP under DSM-5 criteria in patients with OCD and PTSD, these diagnoses were separated in the text of the items.

### Psychometric evaluation (Phase IV)

#### Study design and population

To evaluate the DTAOP in terms of its psychometric performance, a cross-sectional, multicenter observational study was conducted in nine independent specialized (general psychiatric) and highly specialized psychiatric in-and outpatient clinics in The Netherlands.

Between April 2016 and December 2016, a total of 454 adult (aged 18 and older) in-and outpatients with at least one DSM-IV-TR anxiety disorder were evaluated with the DTAOP. Exclusion criteria were: aged younger than 18 years, and no DSM-IV-TR anxiety disorder. The DSM-IV-TR diagnosis was established by administration of the MINI-Plus 5.0.0 [21, 22].

#### Measures

In addition to the DTAOP, the five-level EuroQol five-dimensional questionnaire (EQ-5D-5L) [23] was administered. The EQ-5D-5L is a five-item generic, preference-based self-report measure to describe and value health related quality of life (HRQoL). The EQ-5D-5L contains five domains (mobility, self-care, usual activities, pain/discomfort, and anxiety/depression) and a visual analogue scale (EQ-VAS) for overall health. Each domain is divided into five response options describing the state per domain (no problems, some problems, moderate problems, severe problems, and extreme problems/unable to). An index score can be generated by applying societal preference weights to the health states as completed by the respondent. Based on Dutch tariff, total scores can range from -0.446 to 1 [24], with higher scores indicating better HrQoL. The EQ-VAS is a vertical scale ranging from zero ("worst imaginable health state") to 100 ("best imaginable health state") on which the respondents are asked to rate their overall health.

#### Procedures

Following referral from a primary or other, independent specialized (i.e. secondary) mental health provider, the clinician responsible for intake administered the DTAOP. This was done at the end of the routine intake process, after the diagnostic work-up was completed. The scoring on the DTAOP items, the patients’ demographic variables and two feasibility questions were recorded in anonymized, electronic case report forms. Feasibility was operationalized as the clarity of the total set of items (scored with "Yes" or "No") and the time required to complete the DTAOP. Inter-rater reliability was examined in a random sample of 20% of patients using independent, concurrent DTAOP evaluations performed by a set of two permutable clinicians. After consenting to participate, patients were invited to self-complete the EQ-5D-5L to assess the convergent validity. Criterion validity was evaluated in a random subsample of 50% of patients. In the absence of a validated reference test, the clinical judgment was the reference standard for the evaluation of the criterion validity. Based on a review of the patient’s medical record, two clinicians independently and blinded to the DTAOP score judged whether the patient needed highly specialized psychiatric treatment. An independent researcher verified the agreement between the clinical judgments, and any discrepancies were resolved by discussion between the two clinicians involved. In a three-hour consensus meeting, consortium members reviewed the results of the psychometric analyses and made necessary adjustments to the DTAOP, which resulted in the final version of the DTAOP.

#### Statistical analysis

Demographic and clinical characteristics and feasibility outcomes (clarity and completion time) were analysed by descriptive statistics. Clarity was considered acceptable if ≥90% of the clinicians evaluated the total set of items of the DTAOP as sufficiently clear. The limit of acceptability of the time required to complete the DTAOP was set at ≤10 minutes. Inter-rater reliability was evaluated by the percent agreement and Krippendorff’s alpha [25, 26]. In contrast to the percent agreement, Krippendorff’s alpha takes into account the agreement expected by chance and is invariant to the permutation of observers. For each Krippendorff’s alpha value a 95% bias corrected confidence interval (CI) was generated by 10,000 bootstrap replications. Although clear rules for determining acceptable reliability are lacking, Krippendorff’s alpha values of 0.667 and higher have previously been considered adequate [26]. Following an assessment of data distribution using a Shapiro-Wilk test, Spearman’s rank correlation coefficients between total DTAOP scores and EQ-5D-5L index scores and EQ-VAS scores were computed to assess convergent validity. Correlations of 0.10–0.29 were considered weak, 0.30–0.49 moderate and ≥0.50 strong [27]. Since HRQoL was demonstrated to be sensitive to variations in patient factors [28], the DTAOP was hypothesized to have negative correlations with the EQ-5D-5L index and EQ-VAS. In order to evaluate the criterion of the DTAOP, a receiver-operating characteristic (ROC) curve was constructed. To determine the optimal cut-off score for identifying patients with an anxiety disorder, OCD, or PTSD in need of highly specialized care, Youden indices (J = sensitivity_c_ + specificity_c_ -1) [29] for a range of cut-off scores were generated. To obtain an optimal trade-off between sensitivity and specificity, the cut-off score that achieved the highest Youden index (i.e. the cut-off score that optimized sensitivity and specificity) was selected as the optimal cut-off score. All analyses were conducted using IBM SPSS (Statistical Package for the Social Sciences) version 20.0 (IBM SPSS Statistics for Macintosh, Version 20.0. Armonk, NY: IBM Corp.). Statistical significance was set at P<0.05 (two-tailed).

## Results

### Development

The systematic search identified a total of 4,187 references, of which 34 met the inclusion criteria. Based on the included papers, a list of 46 clinical and sociodemographic indicators of patients with an anxiety disorder, OCD, or PTSD in need for highly specialized care was generated. The PRISMA flowchart of the study selection process and the resulting list of indicators are provided in S2 Appendix. In the brainstorming stage of the concept mapping procedure, 19 additional potential patient indicators were added to the indicators from the systematic review, resulting in a total of 65 indicators of patients with an anxiety disorder, OCD, or PTSD in need of highly specialized care. The resulting concept map revealed eight overarching domains of indicators: 1: treatment course; 2: socio-demographic and personal factors; 3: psychosocial dysfunctioning; 4: psychosocial factors and compensating individual characteristics; 5: psychiatric comorbidity; 6: severity of the anxiety disorder, OCD, or PTSD; 7: suicidal ideation and self-destructive behaviour; and 8: subtypes of OCD. See S3 Appendix for the full list of potential indicators and S4 Appendix for the resulting concept map. Based on the concept map, the initial draft of the DTAOP was generated. See Table 1 for the abbreviated items of the draft version of the DTAOP. An English translation of the full and final DTAOP is provided in S5 Appendix.

**Table 1:** The Decision Tool Anxiety Disorders, OCD and PTSD (DTAOP): Items, response options and scoring system.

DTAOP item^a^		Response options	Score
1	Previous unsuccessful treatment of the current primary diagnosis in specialized care	Yes	1
		No	0
2	Socio-demographic or personal factors maintaining the anxiety disorder, OCD, or PTSD^b^	Yes	1
		No	0
	Example: low IQ, positive family history of anxiety disorders, OCD, or PTSD		
3	Treatment-interfering psychosocial dysfunctioning	Yes	1
		No	0
4	Treatment-interfering psychosocial factors and/or compensating individual characteristics	Yes	1
		No	0
	Example: inadequate social support system, poor illness insight, low motivations, low level of perceived self-efficacy		
5	Treatment-interfering psychiatric comorbidity	Yes	1
		No	0
6	Severe anxiety disorder, OCD, or PTSD	Yes	1
		No	0
7	Acute suicidal ideation and/or self-destructive behaviour	Yes	1
		No	0
8	≥2 subtypes of OCD	Yes	1
		No	0
		No OCD	0

^a^ Item text is abbreviated. See S5 Appendix for an English translation of the full and final DTAOP.

DTAOP = Decision Tool Anxiety Disorders OCD and PTSD; IQ = Intelligence Quotient; Obsessive-Compulsive Disorder (OCD); Post-Traumatic Stress Disorder (PTSD).

### Psychometric evaluation

#### Patient characteristics

Demographic and clinical characteristics of the total sample and its two subsamples are shown in Table 2. The mean (SD) age of the total sample was 35.33 (11.74) years (range = 18–83 years), and 67.2% of the sample was female. The mean total DTAOP score was 3.10 (SD = 1.80, range = 0–7). Mean HRQoL scores as measured by the EQ-5D-5L index and EQ-VAS were 0.50 (SD = 0.27) and 58.55 (SD = 20.28), respectively. Seven patients were excluded from the data analysis because of missing individual DTAOP items (n = 3) or because they were aged younger than 18 years (n = 4). The frequency with which the individual items of the DTAOP were present in the total sample are shown in Table 3.

**Table 2:** Characteristics of the study sample.

	Total sample	Inter-rater reliability sample^a^	Criterion validity sample^a^
N	454	87	216
Age, years			
Mean (SD)	35.33 (11.74)	34.61 (10.88)	34.86 (11.11)
Range	18–83	19–60	18–65
Sex (n, %)			
Male	149 (32.8)	29 (33.3)	66 (30.6)
Female	305 (67.2)	58 (66.7)	150 (69.4)
Diagnosis, n (%)			
GAD/phobia	230 (50.7)	49 (56.3)	91 (42.1)
OCD	137 (30.2)	24 (27.6)	72 (33.3)
PTSD	61 (13.4)	7 (8.0)	37 (17.1)
GAD/phobia and OCD	12 (2.6)	3 (3.4)	6 (2.8)
GAD/phobia and PTSD	12 (2.6)	4 (4.6)	9 (4.2)
GAD/phobia, OCD and PTSD	2 (0.4)	-	1 (0.5)
Total DTAOP score			
Mean (SD)	3.10 (1.80)	3.22 (1.74)	3.38 (1.79)
Range	0–7	0–7	0–7
EQ-5D-5L index			
Mean (SD)	0.50 (0.27)^b^	0.52 (0.27)^d^	0.49 (0.28)^e^
Range	-0.30–1.00	-0.11–1.00	-0.30–1.00
EQ-VAS			
Mean (SD)	58.55 (20.28)^c^	62.71 (19.11)^d^	58.77 (21.20)^f^
Range	0–100	20–95	0–100

DTAOP = Decision Tool Anxiety Disorders, OCD and PTSD; EQ-5D-5L = five-level EuroQol five-dimensional questionnaire; EQ-VAS = EuroQol visual analogue scale; GAD = Generalized Anxiety Disorder; OCD = Obsessive-Compulsive Disorder; PTSD = Post-Traumatic Stress Disorder; SD = Standard Deviation.

^a^ Part of total sample.

^b^ N = 386.

^c^ N = 371.

^d^ N = 78.

^e^ N = 177.

^f^ N = 165.

**Table 3:** Frequency and percentages with which the items of the DTAOP were present in the total sample (n = 454).

DTAOP item^a^		N	%
1	Previous unsuccessful treatment of the current primary diagnosis in specialized care	189	41.6
2	Socio-demographic or personal factors maintaining the anxiety disorder, OCD, or PTSD^b^	186	41.0
3	Treatment-interfering psychosocial dysfunctioning	229	50.4
4	Treatment-interfering psychosocial factors and/or compensating individual characteristics	203	44.7
5	Treatment-interfering psychiatric comorbidity	214	47.1
6	Severe anxiety disorder, OCD, or PTSD	295	65.0
7	Acute suicidal ideation and/or self-destructive behaviour	26	5.7
8	≥2 subtypes of OCD	65	14.3

DTAOP = Decision Tool Anxiety Disorders, OCD and PTSD; OCD = Obsessive-Compulsive Disorder; PTSD = Post-Traumatic Stress Disorder.

#### Feasibility

The average time required to complete the DTAOP was 4.6 minutes (i.e. 4 minutes and 37 seconds) (SD = 2.62, range = 1–20) and the total DTAOP judged as clear in the majority (93.0%) of all evaluations. Nine clinicians expressed concern about the distinctiveness and clarity of item 2 ("Socio-demographic or personal factors maintaining the anxiety disorder, OCD or PTSD") and item 4 ("Treatment-interfering psychosocial factors and/or compensating individual characteristics"). Additionally, eight clinicians suggested the addition of a cut-off score to item 6 ("Severe anxiety disorder, OCD or PTSD") by which the presence or absence of a severe anxiety disorder, OCD or PTSD could be determined. Based on the provided qualitative feedback and further results on the psychometric properties of the DTAOP, consortium members proposed revisions to the wording and instructions of some of the items, resulting in the final version of the DTAOP. See the paragraph “Continued development of the DTAOP” for the description and results of the continued development of the DTAOP.

#### Reliability

As shown in Table 4, the percentage of agreement ranged from 71% to 92%, and Krippendorff’s alpha values ranged from 0.4274 (95% CI, 0.2428–0.6015) for item 2 ("Social factors maintaining the anxiety disorder, OCD or PTSD") to 0.8392 (95% CI, 0.7203–0.9401) for item 1 ("Previous unsuccessful treatment of the current primary diagnosis in specialized care"). The Krippendorff’s alpha values of items 2 to 6 were below the recommended level of 0.667 [26].

**Table 4:** Inter-rater reliability indices as assessed by percent agreement and Krippendorff’s alpha (n = 87).

DTAOP item		% agreement	Krippendorff’s alpha (95% CI)
1	Previous unsuccessful treatment of the current primary diagnosis in specialized care	92	0.8392 (0.7203–0.9401)
2	Socio-demographic or personal factors maintaining the anxiety disorder, OCD or PTSD	71	0.4274 (0.2428–0.6015)
3	Treatment-interfering psychosocial dysfunctioning	82	0.6339 (0.4824–0.7810)
4	Treatment-interfering psychosocial factors and/or compensating individual characteristics	80	0.6114 (0.4432–0.7614)
5	Treatment-interfering psychiatric comorbidity	72	0.4346 (0.2417–0.6106)
6	Severe anxiety disorder, OCD or PTSD	83	0.6235 (0.4541–0.7816)
7	Acute suicidal ideation and/or self-destructive behaviour	98	0.7890 (0.4494–1.0000)
8	≥ 2 subtypes of OCD	84	0.8153 (0.7395–0.8865)

DTAOP = Decision Tool Anxiety Disorders, OCD and PTSD; CI = Confidence Interval.

#### Validity

**Fig 2:**
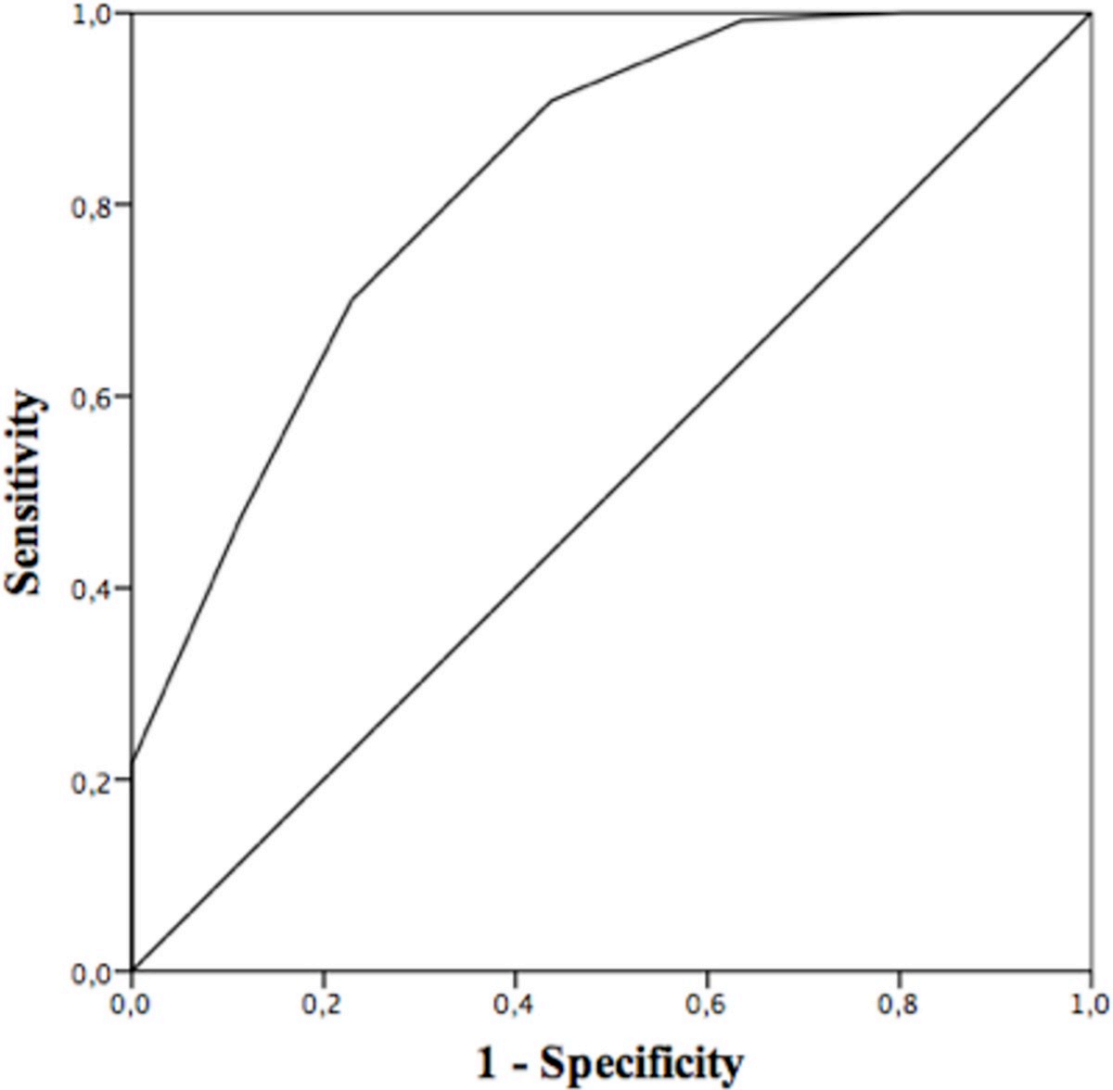
ROC curve for DTAOP (N = 216; AUC = 0.826, 95% CI, 0.772–0.881; P<0.001).

Consistent with our hypotheses, the DTAOP negatively correlated with the EQ-5D-5L index (r_s_(386) = -0.413; P<0.001) and EQ-VAS (r_s_(371) = -0.296; P<0.001). See Fig 2 and Table 5 for the operating characteristics of the DTAOP at various cut-off scores. The area under the curve (AUC) was 0.826 (95% CI, 0.772–0.881; P<0.001) and the Youden index was highest at a cut-off score of ≥4 (J = 0.471), with a sensitivity of 0.700 (95% CI, 0.610–0.780) and a specificity of 0.771 (95%, CI 0.674–0.850).

**Table 5:** Operating characteristics of the DTAOP.

DTAOP scale score	Sensitivity (95% CI)	Specificity (95% CI)	Youden index^a^
≥1	1.000 (0.970–1.000)	0.198 (0.124–0.292)	0.198
≥2	0.992 (0.954–1.000)	0.365 (0.269–0.469)	0.357
≥3	0.908 (0.842–0.953)	0.562 (0.457–0.664)	0.470
≥4	0.700 (0.610–0.780)	0.771 (0.674–0.850)	0.471
≥5	0.475 (0.383–0.568)	0.885 (0.804–0.941)	0.360
≥6	0.217 (0.147–0.301)	1.000 (0.962–1.000)	0.217
≥7	0.017 (0.000–0.030)	1.000 (0.962–1.000)	0.017

DTAOP = Decision Tool Anxiety Disorders, OCD and PTSD; CI = Confidence Interval.

^a^ Youden index = (sensitivity + specificity) - 1.

#### Continued development of the DTAOP

Based on the qualitative feedback (feasibility results) and reliability estimates, consortium members proposed revisions to the wording and instructions of items 2 to 6. To improve the distinctiveness and item clarity of item 2 ("Socio-demographic or personal factors maintaining the anxiety disorder, OCD or PTSD") and 4 ("Treatment-interfering psychosocial factors and/or compensating individual characteristics"), the item wording and instructions of both items were revised. Additionally, cut-off scores by which the presence or absence of treatment-interfering psychosocial dysfunctioning (item 3) and the presence or absence of a severe anxiety disorder, OCD or PTSD (item 6) can be determined were added. Finally, to improve the clarity of item 5 ("Treatment-interfering psychiatric comorbidity"), an item instruction was added by which the presence or absence of a treatment-interfering comorbid psychiatric disorder can be determined. An English translation of the revised and final DTAOP is presented in S5 Appendix. Although the changes are likely to improve item clarity and subsequently enhance item-level inter-rater reliability, future studies are needed to determine the inter-rater reliability of the newly worded items and instructions.

## Discussion

This study presented the development and psychometric evaluation of the Decision Tool Anxiety Disorders, OCD and PTSD (DTAOP). The DTAOP is an eight-item clinician-administered screening measure designed to facilitate the early identification of patients with an anxiety disorder, OCD or PTSD in need of highly specialized care. Scale items were selected in a sequential mixed-methods approach to allow an in-depth exploration of the factors indicating a need for highly specialized care in patients with an anxiety disorder, OCD or PTSD. In this cross-sectional, multicenter observational study in patients with a DSM-IV-TR anxiety disorder, the DTAOP demonstrated excellent feasibility and good validity, but weak inter-rater reliability. To improve the item-level inter-rater reliability, revisions and refinements of the wording and instructions were made, resulting in the final version of the DTAOP.

The study was performed in a sample of patients with a DSM-IV-TR anxiety disorder in routine in-and outpatient treatment to maximize external validity and clinical relevance. The clarity of the total set of items was supported by the majority (93.0%) of respondents and the average completion time was 4 minutes and 37 seconds, indicating that the DTAOP is quick to complete. Despite the satisfactory feasibility results, indicating that scoring of the DTAOP was clear and quick on an individual level, the Krippendorff’s alpha values of five of the items fell short of the minimum recommended reliability level of 0.667 [26]. Although this may be partly due to the use of a highly rigorous measure for assessing inter-rater reliability [30], the qualitative feedback revealed that revisions and refinements of the wording and instructions of the respective items could improve the inter-rater reliability. Although the changes made to the DTAOP were aimed at improving the item-level inter-rater reliability, future studies are needed to confirm this. In addition, previous work has shown that training and frequent use in daily clinical practice can significantly improve scale reliability [31, 32]. Whether training and frequent use also improves DTAOP inter-rater reliability levels, should, however, be subject to future research as well.

Aggregated DTAOP scores demonstrated meaningful patterns of convergent validity with HRQoL scores as measured by the EQ-5D-5L index and EQ-VAS. DTAOP scores were more strongly associated with EQ-5D-5L index scores than with EQ-VAS scores. The stronger association with EQ-5D-5L index scores could be explained by the fact that both the DTAOP and EQ-5D-5L are scored (DTAOP) or valued (index values EQ-5D-5L) by someone other than the patient, which could have reduced effects of for instance coping and adaptation. The DTAOP also demonstrated good criterion validity (AUC = 0.826), indicating that the consensus-based conceptual framework that guided DTAOP development fits the measured construct well.

The main strengths of the present study include the mixed-methods approach used to develop the measure, the large number of examined psychometric properties, and the nationwide representation of participating clinics (nine independent general psychiatric and highly specialized psychiatric in-and outpatient clinics across The Netherlands). Also, to our knowledge, the DTAOP is the first psychometrically validated measure to assess highly specialized care need in patients with an anxiety disorder, OCD, or PTSD. It meets the need for an accurate and easily administrable measure to facilitate the early identification and referral of patients with an anxiety disorder, OCD, or PTSD in need of highly specialized care. However, several limitations of this study should be noted. First, since the present study represents a first cross-sectional evaluation of the psychometric properties of the DTAOP, future studies are needed to replicate and extend these initial findings. More specifically, important areas for future research include the assessment of the inter-rater reliability of the adapted items, the convergent validity with measures of anxiety disorder, OCD, or PTSD severity and psychosocial functioning, the predictive validity for use in clinical and research settings, and the sensitivity to treatment-related change. In addition, since the present study was not powered to detect differences in psychometric performance of the DTAOP between types of DSM-IV-TR anxiety disorders, future research should assess whether DTAOP performs differently in different types of anxiety disorders. Second, it should be noted that the electronic case report forms in which the scoring of the DTAOP was entered by clinicians, did not allow items to be left unanswered. Hence, an evaluation of the feasibility in terms of missing values could not be performed. Third, in the absence of a standard test for the systematic and standardized early identification of patients with a highly specialized mental healthcare need, the clinical judgement clinicians constituted the reference standard for the evaluation of the criterion validity. Although the use of the clinical judgement as the reference standard may have introduced subjective error, effort was made to reduce error by basing the final clinical judgement on dual, independently provided examinations made by two clinicians who were blinded to the index (i.e. DTAOP) score. Fourth, since the aim was to develop an easily administrable measure, the scoring system of the DTAOP was simplified to indicating the ‘absence’ or ‘presence’ of the respective clinical (e.g., suicidal ideation) and non-clinical patient factors (e.g. psychosocial factors). However, it should be noted that measuring clinical and non-clinical patient factors is a complex and nuanced matter. Sensitive and valid assessment of the respective factors, and assessment of their possible treatment-interfering effect (items 3–5), may require a more sensitive approach like Likert or even continuous scoring systems. Likewise, the DTAOP was constructed as an unweighted additive scoring system in order to be easily administrable. Although the use of an unweighted additive scoring system enhances the feasibility (i.e. ease of use) of the DTAOP within the context of daily clinical practice, it diminishes the proportional effect of individual items and possible meaningful interactions between items, and may thereby reduce the validity (i.e. precision) of the resultant classification of patients. However, irrespective of the use of a two-point (dichotomous) scoring system and additive score model of unweighted items, the DTAOP demonstrated to be a valid and clinically applicable operationalization of highly specialized care need. Further work could be carried out to establish the effect of different item-level scoring and the use of a weighted scoring system on the psychometric properties of the DTAOP. Fifth, although the established cut-off score of ≥4 is likely to generalize to specialized and highly specialized care settings in The Netherlands due to the national uniform organisational structure and service delivery of psychiatric services, future studies are needed to establish its cross-national robustness. Sixth, although the DTAOP was initially designed for use in specialized mental healthcare centers to inform “step-up” referral decisions to highly specialized mental healthcare centers, the DTAOP might also inform “step-down” referral decisions from highly specialized care back to specialized mental healthcare care. In addition, use of the DTAOP in primary mental healthcare may further enhance the early identification of patients in need of highly specialized care and the timely selection of the optimal initial treatment in these patients. Future studies are required to evaluate the possible added benefit of such broader use of the DTAOP in primary and highly specialized mental health services. Finally, it should be noted that the DTAOP is not designed to replace careful clinical assessment, but is rather intended to provide probable indications of highly specialized care need and should be used as a first step in a more comprehensive assessment. As such, the DTAOP has the potential to aid in the selection of the most appropriate treatment setting for patients on an individual basis, ultimately benefitting the clinical and cost-effectiveness of treatments.

Despite these limitations, this study provides initial support for psychometric properties of the DTAOP in a sample of patients with a DSM-IV-TR anxiety disorder. The DTAOP demonstrated to be a short and easy scoring, and at a cut-off score of ≥4, valid measure to aid clinicians in the early identification of patients with an anxiety disorder, OCD, or PTSD in need of highly specialized care. Future research is needed to determine the inter-rater reliability of the newly worded items and instructions. Its use in clinical practice will guide in selecting the most appropriate treatment setting, and hence has the potential to benefit treatment outcomes and the efficient use of scarce resources.

## Supporting information

S1 Appendix Search strategy. (PDF)

S2 Appendix PRISMA flow chart. (PDF)

S3 Appendix Concept map clusters and candidate indicators. (PDF)

S4 Appendix Concept map. (PDF)

S5 Appendix Decision Tool Anxiety Disorders, OCD and PTSD (DTAOP). (PDF)

S6 Appendix PRISMA checklist. (PDF)
