# Supplementary material for: Development and psychometric evaluation of the Decision Tool Anxiety Disorders, OCD and PTSD (DTAOP): Facilitating the early detection of patients in need of highly specialized care
Source: PLoS One. 2021 Aug 19;16(8):e0256384. doi: 10.1371/journal.pone.0256384 (PMC8375980; doi:10.1371/journal.pone.0256384)
Supplement: S1 Appendix — (PDF) [file pone.0256384.s001.pdf]

S1 Appendix. Search strategy.

Pubmed (NLM)

#	**Searches**
1	(“anxiety disorders”[MeSH Major Topic] OR “panic disorder”[Mesh major topic] OR “agoraphobia”[Mesh major topic] OR “phobic disorders”[mesh major topic] OR “obsessive-compulsive disorder”[MeSH Major Topic] OR “stress disorders, post-traumatic”[MeSH Major Topic] OR “stress disorders, traumatic, acute”[MeSH Major Topic] OR “hyperventilation”[MeSH Major Topic] OR generalized anxiety disorder[tiab] OR social phobia[tiab]) AND (resistant[ti] OR comorbidity[ti] OR comorbid[ti] OR co-morbidity[ti] OR “co morbidity”[ti] OR “co morbid”[ti] OR severe[ti] OR severity[ti] OR complex[ti] OR complexity[ti] OR non response[ti] OR non-response[ti] OR chronic[ti] OR chronicity[ti] OR recurrent[ti] OR recurring[ti] OR recurrence[ti] OR relapse[ti] OR avoidance[ti] OR admission[ti] OR admissions[ti] OR insight[ti] OR duration[ti] OR “failed treatment”[ti] OR “treatment failure”[ti] OR “patient dropouts”[mesh major topic])
2	limit 1 to full text
3	limit 2 to human
4	limit 3 to english or Dutch language
5	limit 101 to yr="2000 -Current"

PsycINFO (Ovid)

#	**Searches**
1	("Anxiety disorder" or "disorder, anxiety" or "disorders, Anxiety" or "neuroses, Anxiety" or "Anxiety States, Neurotic" or "Anxiety State, Neurotic" or "Neurotic Anxiety State" or "Neurotic Anxiety States" or "State, Neurotic Anxiety" or "States, Neurotic Anxiety").ab,ti.
2	("Disorder, Panic" or "Disorders, Panic" or "Panic Disorders" or "Panic Attacks" or "Attack, Panic" or "Attacks, Panic" or "Panic Attack").ab,ti.
3	Agoraphobia.ab,ti.
4	(Disorder, Phobic or Disorders, Phobic or Phobic Disorder or Phobic Neuroses or Neuroses, Phobic or Phobias or Phobia or Phobia, School or Phobias, School or School Phobia or School Phobias or Claustrophobia or Claustrophobias or Phobia, Social or Phobias, Social or Social Phobia or Social Phobias).ab,ti.
5	(Disorder, Obsessive-Compulsive or Disorders, Obsessive-Compulsive or Obsessive Compulsive

	Disorder or Obsessive-Compulsive Disorders or Neurosis, Obsessive-Compulsive or Neuroses, Obsessive-Compulsive or Neurosis, Obsessive Compulsive or Obsessive-Compulsive Neuroses or Obsessive-Compulsive Neurosis or Anankastic Personality or Anankastic Personalities or Personalities, Anankastic or Personality, Anankastic).ab,ti.
6	(Post-Traumatic Stress Disorder or Stress Disorder, Post-Traumatic or Stress Disorders, Post Traumatic or PTSD or Stress Disorder, Post Traumatic or Neuroses, Posttraumatic or Posttraumatic Neuroses or Posttraumatic Stress Disorders or Posttraumatic Stress Disorder or Stress Disorder, Posttraumatic or Stress Disorders, Posttraumatic or Neuroses, Post-Traumatic or Neuroses, Post Traumatic or Post-Traumatic Neuroses or Post-Traumatic Stress Disorders or Post Traumatic Stress Disorders or Chronic Post-Traumatic Stress Disorder or Chronic Post Traumatic Stress Disorder or Delayed Onset Post-Traumatic Stress Disorder or Delayed Onset Post Traumatic Stress Disorder or Acute Post-Traumatic Stress Disorder or Acute Post Traumatic Stress Disorder).ab,ti.
7	(Stress Disorders, Acute or Acute Stress Disorder or Stress Disorder, Acute or Acute Stress Disorders).ab,ti.
8	Hyperventilation.ab,ti.
9	1 or 2 or 3 or 4 or 5 or 6 or 7 or 8
10	resistant.ti.
11	Comorbidity.ti.
12	Comorbid.ti.
13	Co-morbidity.ti.
14	Co morbidity.ti.
15	co morbid.ti.
16	Severe.ti.
17	Severity.ti.
18	Complex.ti.
19	Complexity.ti.
20	Non response.ti.
21	Non-response.ti.
22	Chronic.ti.
23	Chronicity.ti.
24	Recurrent.ti.
25	Recurring.ti.

26	Recurrence.ti.
27	Relapse.ti.
28	Avoidance.ti.
29	Admission.ti.
30	Admissions.ti.
31	Insight.ti.
32	Duration.ti.
33	failed treatment.ti.
34	treatment failure.ti.
35	(Dropout, Patient or Dropouts, Patient or Patient Dropout or Dropout Characteristics or Characteristic, Dropout or Characteristics, Dropout or Dropout Characteristic or Dropouts or Dropout).ab,ti.
36	10 or 11 or 12 or 13 or 14 or 15 or 16 or 17 or 18 or 19 or 20 or 21 or 22 or 23 or 24 or 25 or 26 or 27 or 28 or 29 or 30 or 31 or 32 or 33 or 34 or 35
37	9 and 36
38	Limit 37 to full text
39	Limit 38 to human
40	Limit 39 to dutch or english language
41	Limit 40 to yr="2000 -Current")
