# Supplementary figures and images for: Development and psychometric evaluation of the Decision Tool Anxiety Disorders, OCD and PTSD (DTAOP): Facilitating the early detection of patients in need of highly specialized care
Source: PLoS One. 2021 Aug 19;16(8):e0256384. doi: 10.1371/journal.pone.0256384 (PMC8375980; doi:10.1371/journal.pone.0256384)

## S2 Appendix. PRISMA flow chart.

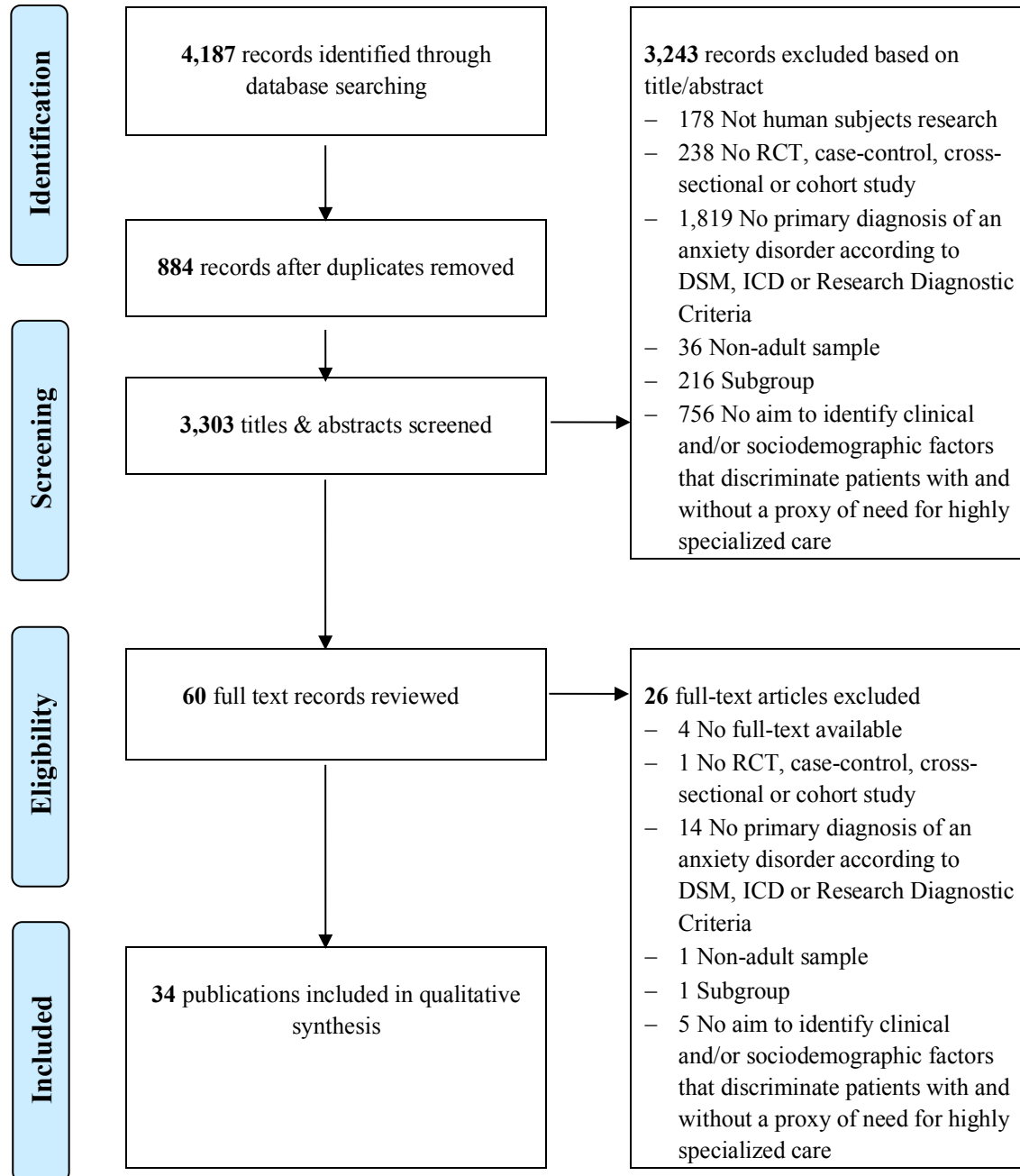

Supplement: S2 Appendix — (PDF) [file pone.0256384.s002.pdf]
