# Supplementary material for: Development and psychometric evaluation of the Decision Tool Anxiety Disorders, OCD and PTSD (DTAOP): Facilitating the early detection of patients in need of highly specialized care
Source: PLoS One. 2021 Aug 19;16(8):e0256384. doi: 10.1371/journal.pone.0256384 (PMC8375980; doi:10.1371/journal.pone.0256384)
Supplement: S3 Appendix — (PDF) [file pone.0256384.s003.pdf]

S3 Appendix. Concept map clusters and candidate indicators.

Cluster		Indicator	Systematic Review[a]	Brainstorm[b]
**1. Treatment course**				
	1.	>1 time relapse		X
	15.	Repeated treatments without remission		X
	17.	Treatment resistant		X
	33.	Partial remission	X	
	35.	Earlier onset age	X	
	45.	No current pharmacological treatment	X	
	51.	Chronic course	X	
	56.	Higher level of pretreatment symptoms	X	
**2. Socio-demographic and personal factors**				
	11.	Lower intellectual functioning		X
	14.	Low level of motivation		X
	24.	Younger age	X	
	34.	Having no partner	X	
	37.	Female	X	
	47.	Higher self-transcendence score	X	
	48.	Fewer years of education	X	
	52.	OCD in first-degree relatives	X	
	57.	Perceived criticism on patient from family members	X	
	60.	Perceived criticism	X	
**3. Psychosocial dysfunctioning**				
	2.	Worse functioning		X
	5.	Extensive consequential damages		X
	12.	Severe stagnation in multiple life domains		X
	25.	Disabilities in physical functioning		X
	31.	Level of functioning	X	
	43.	Unemployment	X	
	63.	Inability to work due to illness	X	
**4. Psycho-social factors and compensating individual**				
	4.	Low self-efficacy		X
	10.	High tendency to avoid anxiety		X
	13.	Lack of compensating competencies		X
	29.	Stressful life events	X	
	55.	Psychosocial difficulties	X	
	59.	Poor insight	X	
	64.	Less vitality	X	

**5. Psychiatric comorbidity**			
3.	Multiple comorbid diagnoses		X
6.	OCD with comorbid TIC-disorders		X
9.	Less common anxiety complaints such as conversion		X
18.	Severe eating disorder		X
19.	Bodyd dismorphic disorder		
20.	Comorbid severe depression	X	
21.	Comorbid personality disorder	X	
28.	Comorbid anxiety disorder	X	
41.	Avoidant personality disorder	X	
49.	Bipolar disorder	X	
53.	Obsessive-compulsive personality disorder	X	
54.	Comorbid alcohol or other substance use disorder	X	
**7. Severity**			
7.	High level of distress		X
22.	Severe OCD	X	
27.	Severe anxiety symptoms	X	
30.	Severity of complaints	X	
32.	Level of neuroticism	X	
36.	Severity of avoidance in PTSD	X	
39.	Severity of PTSD	X	
44.	Severity of hoarding	X	
50.	Higher level of arousal	X	
61.	Higher severity of anxiety	X	
**8. Suicidal risk**			
8.	Presence of suicidal risks		X
16.	Severe self-destructive behaviour		X
26.	Severity of childhood trauma	X	
46.	History of self-harm	X	
**9. OCD subtypes**			
23.	Contamination fears and washing compulsions	X	
38.	Aggressive obsessions	X	
40.	Higher number of OCD-subtypes	X	
42.	Forbidden thoughts (sexual, religious and aggressive obsessions)	X	
58.	Somatic obsessions	X	
62.	Severe depression	X	
65.	Higher level of physical aggression	X	
**Total**		**46**	**19**

[a] Indicators identified in the systematic review (Phase I).
[b] Indicators generated in the brainstorming stage of the concept mapping procedure (Phase II).
