# Supplementary material for: Development and psychometric evaluation of the Decision Tool Anxiety Disorders, OCD and PTSD (DTAOP): Facilitating the early detection of patients in need of highly specialized care
Source: PLoS One. 2021 Aug 19;16(8):e0256384. doi: 10.1371/journal.pone.0256384 (PMC8375980; doi:10.1371/journal.pone.0256384)
Supplement: S4 Appendix — (PDF) [file pone.0256384.s004.pdf]

S4 Appendix. Concept map.

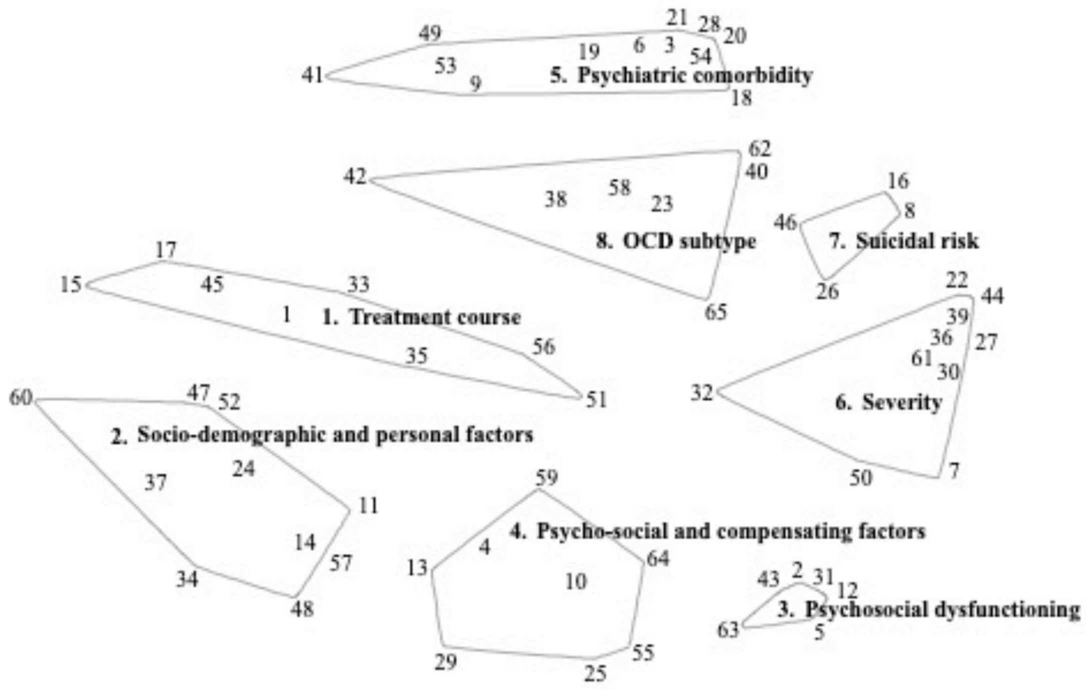

Figure. Concept map of the eight overarching domains of patients with an anxiety disorder in need of highly specialised care (stress value = .298). The numbers correspond to the indicators that were sorted into each category (see Appendix C for an overview of the indicators). Indicators that are closer together indicate higher degrees of similarity based on sorting.
