# Supplementary material for: Development and psychometric evaluation of the Decision Tool Anxiety Disorders, OCD and PTSD (DTAOP): Facilitating the early detection of patients in need of highly specialized care
Source: PLoS One. 2021 Aug 19;16(8):e0256384. doi: 10.1371/journal.pone.0256384 (PMC8375980; doi:10.1371/journal.pone.0256384)
Supplement: S5 Appendix — (PDF) [file pone.0256384.s005.pdf]

Decision Tool Anxiety Disorders, OCD and PTSD (DTAOP)

V21.01

Name of patient:

Date:

Name of clinician:

		Comment
1. Have there been any unsuccessful treatments in specialized mental healthcare for the primary diagnoses?	○ yes<br>○ no	
2. Are there any social factors contributing to the anxiety disorder, OCD, or PTSD that are hard to influence?<br>Note: Also think of low education, unemployment, little or no support system, and a dysfunctioning family system.	○ yes<br>○ no	
3. Does the patient exhibit severe psychosocial dysfunctioning that interferes with the anxiety, OCD, or PTSD treatment?<br>• Note:<br>• GAF≤50 or WHODAS≥130 is an indication of severe dysfunctioning.<br>• There is interference if the degree of psychosocial dysfunctioning complicates the clinical presentation of the primary diagnosis, or the treatment of the primary diagnosis.	○ yes<br>○ no	
4. Does the patient have a disadaptive coping style that interferes with the anxiety, OCD, or PTSD treatment?<br>Note: Think of low motivation, lack of compensating individual characteristics, and a low level of perceived self-efficacy.	○ yes<br>○ no	
5. Does the patient have at least one diagnosed comorbid psychiatric disorder that interferes with the anxiety, OCD, or PTSD treatment?<br>Note:<br>• Also think of personality disorders, development disorders, addiction, and intellectual disabilities.<br>• There is interference if the diagnosed comorbid disorder complicates the clinical presentation of the primary diagnosis, or the treatment of the primary diagnosis.	○ yes<br>○ no	
6. Does the patient have a severe anxiety disorder, OCD, or PTSD?<br>Note: besides a clinical impression of severity does the patient score high on one of the following measures:<br>• General measures: SCL-90 high or very high in comparison to a normative sample of outpatients; BSI high or very high in comparison to a normative sample of outpatients.<br>• Anxiety disorder: BAI ≥26.<br>• OCD: Y-BOCS ≥24; diminished/no sense of reality.<br>• PTSD: CAPS-5 average item score >3; PCL-5 average item score >3; DSM-5 severe.	○ yes<br>○ no	
7. Does the patient have acute suicidal ideation and/or self-destructive behaviour?	○ yes<br>○ no	

P.T.O.

Decision Tool Anxiety Disorders, OCD and PTSD (DTAOP)

8. In case of OCD, are there 2 or more subtypes present?
   Examples of OCD subtypes are:
   - Compulsive washing
   - Compulsive checking
   - Compulsive hoarding
   - Obsession with symmetry, ordering/arranging, or counting
   - Aggressive, religious, or sexual intrusion

○ yes
○ no
○ no OCD

Comment

Total amount of positive (=yes) scores ≥ 4?

Yes → indicated for highly specialized care on the basis of the DTAOP
No → not indicated for highly specialized care on the basis of the DTAOP

© 2017 TOPGGz and iMTA All rights reserved. The DTAOP has been released for use and may be copied. It is not allowed to change the text. For questions or for reporting experiences with the decision tool, please email us at info@topggz.nl
